# Structural consequences of sequence variation in mammalian prion β2α2 loop segments

**DOI:** 10.3389/fnins.2022.960322

**Published:** 2022-10-20

**Authors:** Calina Glynn, Evelyn Hernandez, Marcus Gallagher-Jones, Jennifer Miao, Christina J. Sigurdson, Jose A. Rodriguez

**Affiliations:** ^1^Department of Chemistry and Biochemistry, STROBE NSF Science and Technology Center, UCLA-DOE Institute for Genomics and Proteomics, University of California, Los Angeles, Los Angeles, CA, United States; ^2^Department of Pathology and Medicine, University of California, San Diego, La Jolla, CA, United States

**Keywords:** prion, structure, transmission, cryoEM, microED

## Abstract

Sequence variation in the β2α2 loop, residues 165-175 of the mammalian prion protein (PrP), influences its structure. To better understand the consequences of sequence variation in this region of the protein, we biochemically and biophysically interrogate natural and artificial sequence variants of the β2α2 loop of mammalian PrP. Using microcrystal electron diffraction (MicroED), we determine atomic resolution structures of segments encompassing residues 168-176 from the β2α2 loop of PrP with sequences corresponding to human, mouse/cow, bank vole/hamster, rabbit/pig/guinea pig, and naked mole rat (elk-T174S) β2α2 loops, as well as synthetic β2α2 loop sequences. This collection of structures presents two dominant amyloid packing polymorphisms. In the first polymorph, denoted “clasped”, side chains within a sheet form polar clasps by facing each other *on the same strand*, exemplified by the mouse/cow, human, and bank vole/hamster sequences. Because its stability is derived from within a strand and through polar ladders within a sheet, the sequence requirements for the mating strand are less restrictive. A second polymorph, denoted “interdigitated,” has sidechains interdigitate across mating sheets, exemplified by the elk, naked mole rat (elk T174S), and rabbit sequences. The two types of packing present distinct networks of stabilizing hydrogen bonds. The identity of residue 174 appears to strongly influence the packing adopted in these peptides, but consideration of the overall sequence of a given segment is needed to understand the stability of its assemblies. Incorporation of these β2α2 loop sequences into an 85 residue recombinant segment encoding wild-type bank vole PrP^94–178^ demonstrates that even single residue substitutions could impact fibril morphology as evaluated by negative stain electron microscopy. This is in line with recent findings supporting the accessibility of different structural geometries by varied mammalian prion sequences, and indicates that sequence-specific polymorphisms may be influenced by residues in the β2α2 loop.

## Introduction

Pathogenic prions cause neurodegenerative diseases with varying clinical presentation across mammals in a sequence and strain dependent manner (Fraser and Dickinson, 1973; Aguzzi et al., 2007). Prion species barriers can be eliminated if the animal being infected has a prion protein (PrP) amino acid sequence that matches the inoculum PrP sequence (Scott et al., 1989; Prusiner et al., 1990). This match of PrP^Sc^ to host sequence thus controls the clinical signs, survival time, and brain regions targeted in a manner where the resulting aggregate is not simply a biproduct of infection (Prusiner and DeArmond, 1994; Wang et al., 2010; Foliaki et al., 2018). While the PrP sequence is highly conserved across mammals, a small number of sites along the sequence display variability. Two regions of relatively high variability are distant in sequence (residues 165-175 and 215-223) but proximal in the structure of native cellular PrP (PrP^C^) (Billeter et al., 1997) and in some strains of PrP^Sc^ (Hoyt et al., 2021; Kraus et al., 2021; Manka et al., 2021, 2022). Structural studies of PrP^C^ from various mammals have illuminated a conserved fold containing an ordered globular domain with three alpha helices and two beta strands (Riek et al., 1996). A site of particular interest in the native structure of PrP is the loop formed by residues 165-175 (Gossert et al., 2005), referred to as the β2α2 loop. The structural rigidity of the β2α2 loop in PrP^C^ has been proposed to influence prion behavior, by means of single residue substitutions at two positions: 170 and 174. Disease transmission is facilitated when host and inoculum sequences match at residues 170 and 174, despite sequence variation at other positions (Sigurdson et al., 2010).

Given the influence of sequence identity on the structural state of the β2α2 loop in PrP^C^, its local structure in the context of PrP^Sc^ is of great interest. The β2α2 loop of mammalian prions can be highly amyloidogenic in isolation, with β2α2 loop segments as short as six residues readily forming amyloid crystals (Sawaya et al., 2007; Wiltzius et al., 2009; Rodriguez et al., 2017). The structures of human (^170^SNQNNF^175^) (Sawaya et al., 2007) and elk (^170^NNQNTF^175^) (Wiltzius et al., 2009) β2α2 loops pack into distinct amyloid structures. Likewise, a recently determined microcrystal electron diffraction (microED) structure of a nine residue segment of the bank vole β2α2 loop adopts a compact amyloid fold in which polar residues form stabilizing networks of hydrogen bonds (Gallagher-Jones et al., 2018). Compared to the previously determined 6-mer structures, the latter also illuminates contacts made by key residues 168 and 169 (Kurt et al., 2014, 2015; Leske et al., 2017).

To gain insights into the influence of sequence variation at the β2α2 loop on the amyloid state of PrP segments, we structurally and biophysically characterized a collection of amyloid-forming peptides encompassing nine-residue segments of mammalian prion β2α2 loops. These segments span residues 168-176 (human numbering) and represent PrP loops from species with varying susceptibility to prion disease. We found that the identity of residue 174 influenced the adoption of two distinct amyloid packing polymorphs. One polymorph was stabilized primarily by polar clasps within the same chain, while a second is stabilized by interdigitating sidechains reaching toward a mated strand. However, the identity of residue 174 did not alone dictate the biophysical behavior of β2α2 loop segments in the amyloid state. Instead, the identities of polar residues across the β2α2 loop PrP segments seem to concertedly influence their amyloid state.

## Results

### Microcrystal electron diffraction structures of amyloids formed by the β2α2 loop of naturally occurring mammalian prions

**FIGURE 1:**
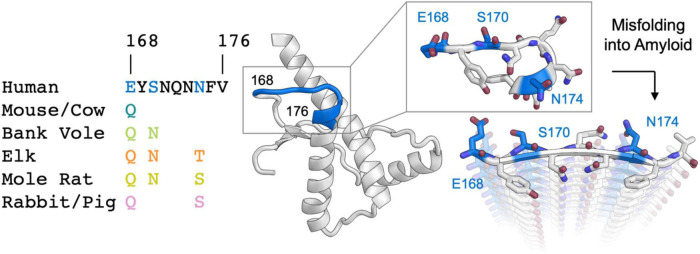
Analysis of sequence variability in polar residues of the β2α2 loop of the mammalian prion protein. The β2α2 loop of mammalian prions **(left)** takes on a loop structure in PrP^C^ **(center and top right)** but misfolds into a beta sheet amyloid conformation **(bottom right)**. Residues that vary between mammals (168, 170, and 174) are colored. Human PrP^C^ structure with PDB ID 1QLX (Zahn et al., 2000) is shown.

**FIGURE 2:**
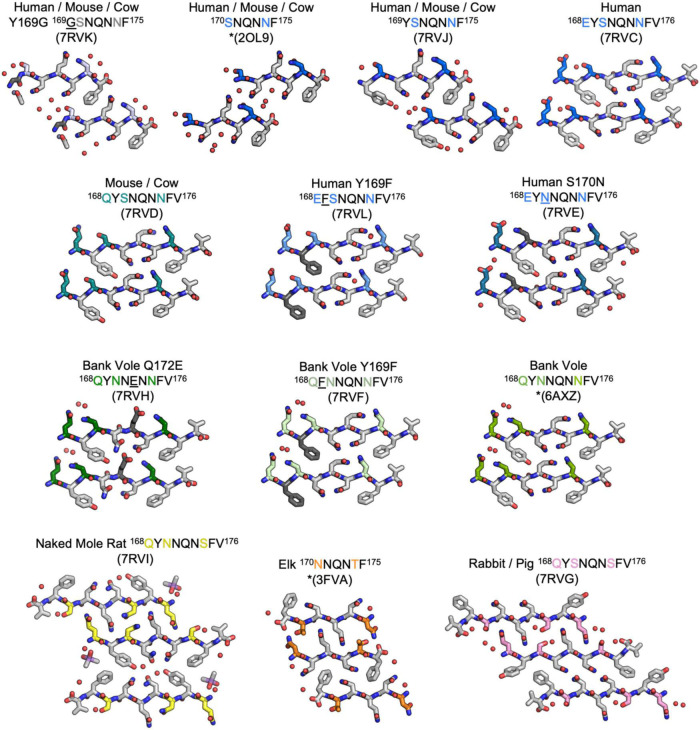
Structures of crystalline mammalian prion β2α2 loop aggregates. A single pair of strands is shown for each 9-residue segment to illustrate the interface between protofilaments. Additionally, three published structures from peptides SNQNNF (Sawaya et al., 2007), NNQNTF (Wiltzius et al., 2009), and QYNNQNNFV (Gallagher-Jones et al., 2018) (starred) are included for reference. All structures except for the bottom row are class 2 (face-to-back) steric zippers that fall into packing category 1. The bottom row is composed of class 1 (face-to-face) steric zippers that fall into packing category 2.

To better understand the amyloid conformations adopted by segments of the β2α2 loop of mammalian prions, we compared the atomic resolution crystal structures formed by β2α2 loop peptides. Our study of these segments was inspired by a previous structure of the bank vole β2α2 loop segment (Gallagher-Jones et al., 2018). These segments consisted of β2α2 loop sequences found in human, mouse/cow, pig/rabbit, and naked mole rat PrP as well as non-native sequences containing one or more substitutions at positions 168, 169, and 172 (Figures 1, 2). Purified peptides were induced to assemble crystals under various conditions (Table 1), with all crystals displaying needle-like morphologies of varying sizes. Crystal suspensions were deposited onto electron microscopy grids, blotted, and plunge frozen for inspection in an electron microscope. Diffraction was collected from one or more crystals to generate high-completeness, high-resolution datasets suitable for structure determination by direct methods. All structures revealed tightly packed beta strands (Figure 2) stacked ∼4.8Å apart with sheets packed parallel and in-register.

**TABLE 1:** Crystallization conditions for β2α2 loop peptides.

Species (Mutation)	Sequence	Concentration (mg/ml)	Solute	Filtered	Crystallization Condition
Human	EYSNQNNFV	4	Water	No	20% EtOH 0.1M NaAc pH 4.5 0.1M Li_2_SO_4_
Mouse/Cow	QYSNQNNFV	10	Water	No	15% EtOH 0.2M MES pH 6.0 0.5M ZnOAc
Naked mole rat	QYNNQNSFV	4	1% DMSO	Yes	5% isopropanol 0.1M NaCacodylate pH 6.5 0.1M ZnAc
Rabbit/Pig	QYSNQNSFV	2	4% DMSO	Yes	0.05M CHES pH 9 0.2M Li_2_SO_4_ 1.3M Na/K Tartrate
Human (S170N)	EYNNQNNFV	2	Water	Yes	0.1M NaOAc pH 4.5 1M NaCl 0.1M Li_2_SO_4_
Human (Y169F)	EFSNQNNFV	2	Water	Yes	Water
Bank Vole (Y169F)	QFNNQNNFV	2	Water	No	10% MPD 0.2M MES pH 6
Bank Vole (Q172E)	QYNNENNFV	1	Water	Yes	0.1M NaAc pH 4.5 2.5M NaCl 0.1M Li_2_SO_4_
Human/Mouse (Y169G)	GSNQNNF	10	Water	Yes	10% (w/v) PEG-8000 0.1M MES pH 6 0.2M Zn(OAc)_2_
Human/Mouse/Cow	YSNQNNF	10	Water	Yes	10% (w/v) PEG-3000; 0.1M Na/K phosphate pH 6.2

**FIGURE 3:**
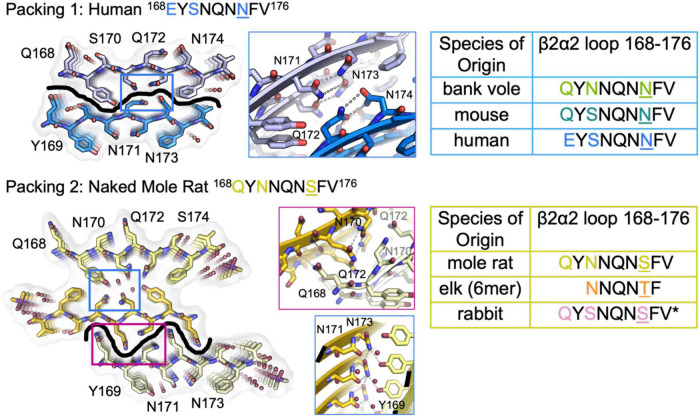
β2α2 Loop Structures Fall Into Two Structural Categories. Packing arrangement for mouse, human, and bank vole **(top)** and mole rat, elk, and rabbit **(bottom)** β2α2 loops with black line highlighting the promiscuous **(top)** and selective **(bottom)** interfaces formed in each packing category. Insets show a conserved hydrogen bonding network across species between N171 and N173 (center column, blue boxes). For bank vole, mouse, and human loops **(top)** N172 and N174 form a clasp (center column, top blue box) while elk and naked mole rat structures **(bottom)** form a clasp between N170 and N172 (center column, center pink box), leaving Q168 free to form a polar ladder that can interdigitate with a complementary mated sheet. Residues that vary between species are colored in text and residue 174 is shown in color and underlined. Bound waters and ligands shown adjacent to their nearest peptide chain.

The refined atomic structures determined from these segments could be classified into two categories (Figure 3): Peptides encoding bank vole/hamster, mouse/cow, and human sequences formed similar structures stabilized primarily by polar clasps (clasped zippers). Those derived from elk and naked mole rat formed structures with extended interdigitating residues across sheets (canonical zippers). Residues in structures from each of these two categories adopt distinct arrangements with specific hydrogen bond networks (Figures 2, 3). A distinguishing feature across the two groups was the identity of residue 174 (clasped: N; interdigitated: T or S); the structures were otherwise insensitive to certain other polar residue substitutions (Figure 2).

### Features of clasped structures formed by wild type β2α2 loop segments

**FIGURE 4:**
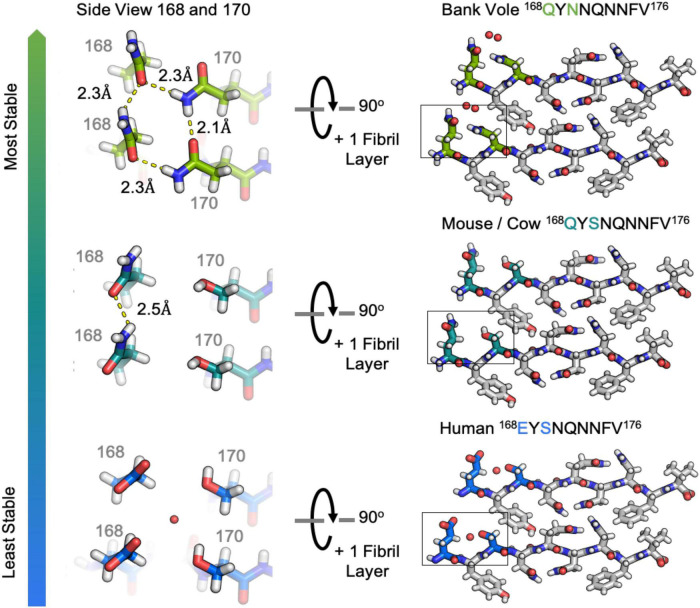
Hydrogen bonding in clasped structures of segments from the β2α2 Loop. Structures from the amyloid state of bank vole **(top)** mouse **(center)** and human **(bottom)** β2α2 loops show a conserved interface and sets of hydrogen bond networks **(right)**. Species-specific differences in the hydrogen bonding networks between residues 168 and 170 give rise to differing stabilities for the three crystalline aggregates **(left)**.

The clasped structures formed by mouse/cow, human, and bank vole/hamster β2α2 loop segments display hydrogen bonding networks that link polar residues between and within strands in a sheet (Figure 3). While their residues adopt overall similar conformations, their chemical identities are expected to dictate the stability of their assemblies across different environments. For example, a glutamate at position 168 in the human structure limits polar ladder – and crystal – formation at neutral or higher pH (Figure 4). Likewise, since human residue S170 cannot form polar ladders and E168 is prohibited from doing so at physiological pH, aggregation of human β2α2 loop segments is more likely to be allowed in acidic environments. This behavior is also observed in full length recombinant human PrP fibrils, for which a low pH environment promotes fibrillization (Swietnicki et al., 2000). In contrast, the structure formed by the mouse/cow segment displays a stabilizing polar ladder formed by glutamine residues at position 168 along the amyloid spine (Figure 4); this network is not anticipated to be affected by neutral pH. The structure formed by the bank vole/hamster segment appears to be the most stable of the group, with 4 additional hydrogen bonds linking residues 168 and 170 in a polar clasp both within a sheet and down the fibril axis (Figure 4).

### Features of interdigitated structures formed by wild type β2α2 loop segments

In interdigitated structures like those formed by naked mole rat, rabbit, and elk segments, hydrogen bond networks normally orchestrated by N174 are disrupted. When it is replaced by threonine in elk and serine in naked mole rat, neither T174 nor S174 can form a polar clasp with residue Q172, and a new network between 170 and 172 is formed that influences hydrogen bonding throughout the structure (Figure 3). Q168 in the naked mole rat structure does not participate in a polar clasp, as it does in its mouse/cow or bank vole/hamster counterparts. Instead, it forms a polar ladder stabilized by interdigitation with Q172 on an opposing chain. This kind of inter-sheet stabilization is observed in both naked mole rat and rabbit β2α2 loop segment structures and in a previously published structure of an elk segment spanning residues 170-175 (Apostol et al., 2010).

**FIGURE 5:**
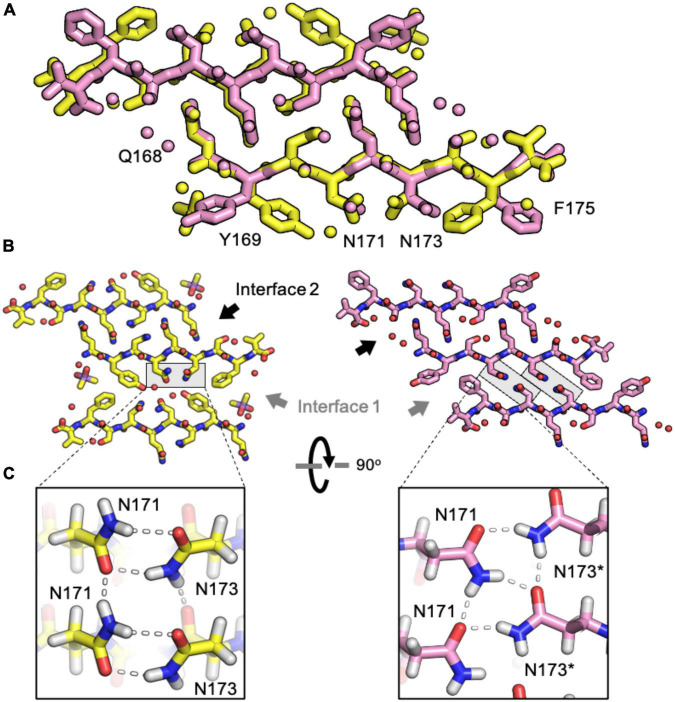
Comparison of naked mole rat and rabbit loop structures. **(A)** Naked mole rat (yellow) and rabbit (pink) loop structures share a common interface seen in packing category 2. **(B)** Interface 1 is composed of inward facing aromatic residues and a polar clasp between N171 and N173 on the same strand for all structures regardless of packing category with the exception of the rabbit loop structure (right). **(C)** A polar clasp is formed in both non-rabbit (left) and rabbit (right) structures, however, in the rabbit structure the clasp is formed by N171 and N173 on mated sheets rather than via interactions within a single sheet.

The rabbit/pig β2α2 segment harbors two serine substitutions, S170 and S174, an unusual combination that gives rise to a rigid loop in PrP^C^ (Wen et al., 2010). Because of the pronounced impact of these residues on the native PrP structure, we investigated their impact on its amyloid conformation. Like the naked mole rat β2α2 loop structure, the rabbit structure displays two unique steric zipper interfaces and interdigitation of polar ladders (Figure 5). The interface harboring interactions between even numbered residues (interface 2) is similar across the rabbit and naked mole rat structures. In contrast, the opposing interface (interface 1) is unique in the rabbit structure; different from all other β2α2 segment structures (Figure 5). In the rabbit structure, N171 and N173 still form polar clasps, but instead of stabilizing residues on the same strand, the residues reach across toward a mating sheet and pair with a residue on the opposing strand. In consequence, its aromatic residues face away from its polar core.

**FIGURE 6:**
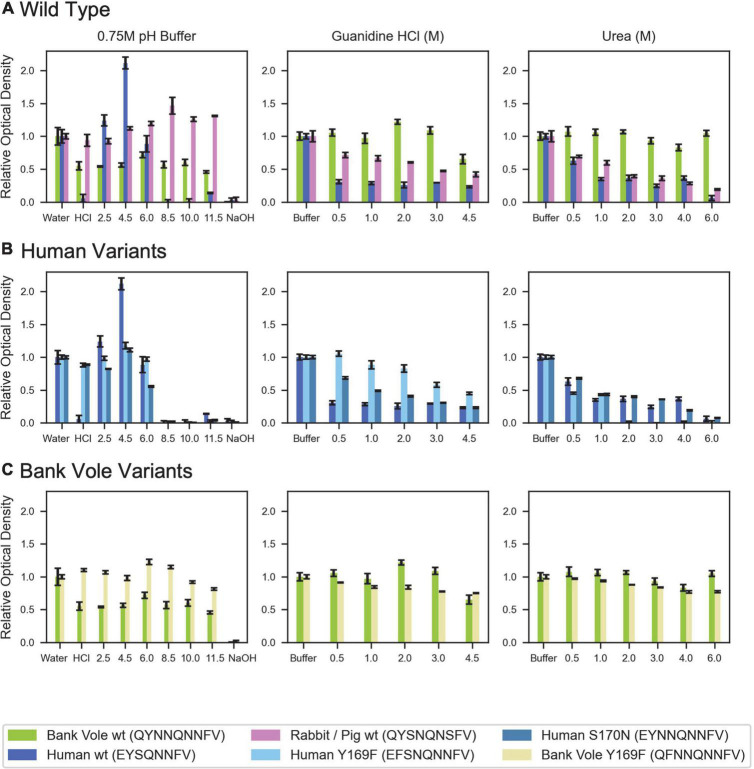
Denaturation of β2α2 loop crystals. **(A)** Wild type peptide crystals were subjected to 0.75M buffers at the specified pH (left column), increasing concentrations of guanidine HCl (center column), and increasing concentrations of urea (right column). **(B)** Human and **(C)** bank vole variants, including the wild type crystals shown in **(A)**, plotted on the same axis as their wild type counterparts for direct comparison. All experiments were performed in triplicate; error bars correspond to standard deviation of the measurements.

While structurally similar, the clasped structures formed by human and bank vole β2α2 loop sequences varied in their relative stabilities in the face of chemical denaturants or pH changes (Figure 6). Likewise, their stabilities differed from those of structures formed by the rabbit segment and those of single residue variants of human and bank vole segments, with structures formed by the rabbit β2α2 loop segment being most resistant to elevated pH (Figure 6).

### Microcrystal electron diffraction structures from β2α2 segments with unnatural sequence variations

To assess the influence of individual residues on the structure and stability of β2α2 loop aggregates, we characterized segments with non-natural sequences. The S170N mutation is one of two residues involved in forming the “rigid loop” seen in some mammalian PrP^C^ structures (Sigurdson et al., 2010; Kurt et al., 2015). Also noteworthy, sequence variation at Y169 is known to affect protease-resistance of the resulting prion aggregates (Kurt et al., 2014; Leske et al., 2017). A Y169G mutation completely hinders disease transmission (Kurt et al., 2014) while a Y169F mutation has no impact on infectivity, but yields increased protease sensitivity (Leske et al., 2017). We assessed the impact of these mutations on structure and stability through three variants: human S170N and Y169F, and bank vole Y169F. The structure adopted by a 7 residue variant with the Y169G mutation (^169^GSNQNNF^175^) has been explored in other work (Martynowycz et al., 2017; Hattne et al., 2018) and a structure adopted by a synthetic bank vole Q172E mutant - ^168^QYNNENNFV^176^ (Figure 2) has likewise been described (Richards et al., 2021). We found that none of these substitutions induced substantial alteration to their parent structures. Thus, the effects induced by these variants may not be fully captured by end-point peptide crystal structures.

**FIGURE 7:**
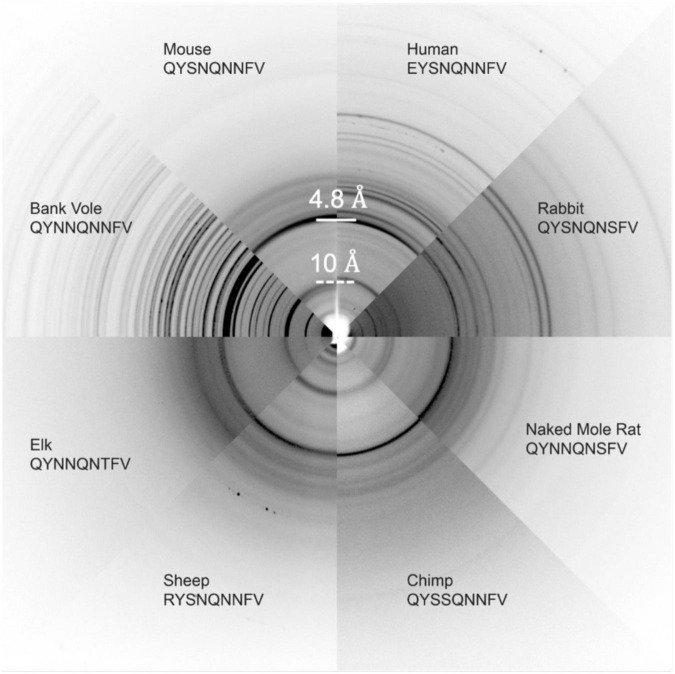
Fiber Diffraction of β2α2 loop aggregates. Fiber diffraction patterns were collected for crystalline (bank vole, mouse, human, rabbit, and naked mole rat) and fibrillar (chimp, sheep R168 sequence polymorph, and elk) aggregates. All aggregates share ∼4.8 and ∼10Å reflections indicative of underlying amyloid structure.

Several other nine-residue β2α2 loop segments of interest, including those representing chimp (QYSSQNNFV), elk (QYNNQNTFV), and the disease preventing sheep Q168R sequence polymorphism (RYSNQNNFV), were recalcitrant to crystallization, even at the nanoscale. For these samples, fiber diffraction was used to assess the amyloid character of the resulting aggregates. All aggregates, regardless of whether they were crystalline or fibrillar in structure, displayed canonical features of amyloid steric zippers (Figure 7) including fiber diffraction patterns with reflections at approximately 4.7-4.8Å and 10Å, indicative of the inter-strand and inter-sheet spacings, respectively.

### Influence of sequence variation in the β2α2 loop on the morphology of bank vole prion fibrils

**FIGURE 8:**
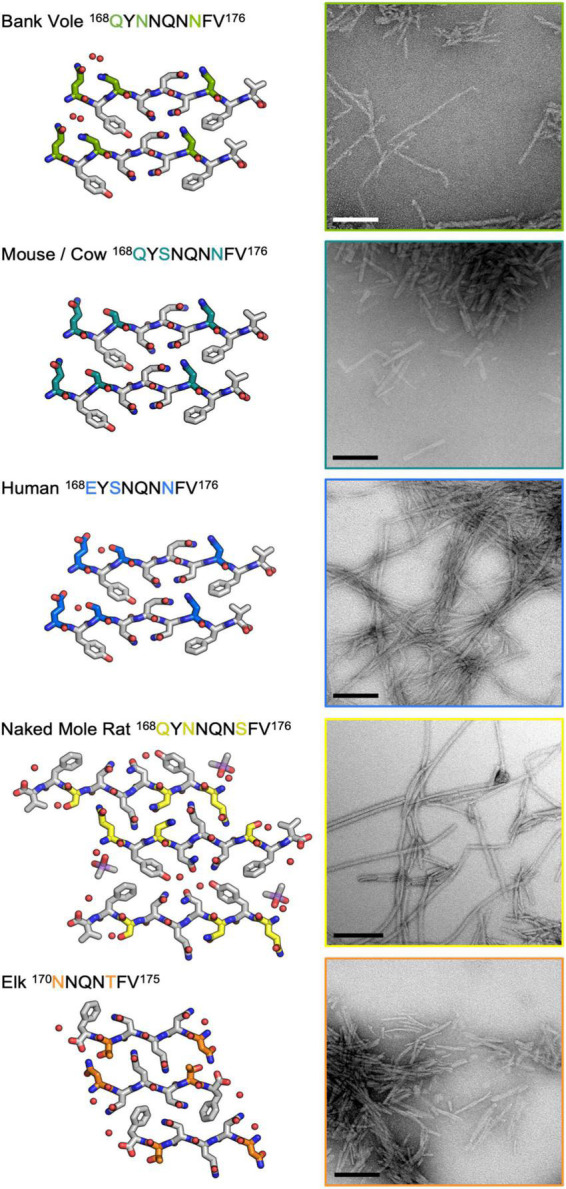
Influence of the β2α2 loop on fibril morphology. Crystal structures for bank vole, mouse/cow, human, naked mole rat, and elk β2α2 loops are shown along with fibrils formed by rBvPrP^94– 178^ harboring the loop substitutions in residues 168-176 belonging to each species. Scalebars = 200nm.

To further assess the impact of β2α2 loop sequence variants, we explored the consequences of variability at polar sites within the β2α2 loop in PrP fibrils. Substitutions were introduced into the bank vole PrP^94–178^ sequence; this segment included the β2α2 loop, all residues previously believed to be part of a beta solenoid prion core (Govaerts et al., 2004), and all residues found in the ordered cores of two prion constructs thoroughly characterized by mouse experiments (Choi et al., 2016), ssNMR (Theint et al., 2017), or cryo-EM (Glynn et al., 2020; Li et al., 2021). β2α2 loop residues in the bank vole sequence were altered to match elk, naked mole rat, human, and mouse/cow sequences. All constructs were allowed to assemble into fibrils under identical growth conditions, such that differences in morphology would not result from discrepancies in chemical environment. Instead, morphological differences would be due to differences in the properties of each sequence. The fibrils formed by the variants displayed distinct morphologies as assessed by negative stain EM (Figure 8). Fibrils containing the native bank vole loop sequence showed rapid twisting with crossover distances of approximately 27 nm, while mouse loop containing fibrils formed very short thick fibrils, human loop formed long matted fibrils, elk loop formed short fibrils of mixed thickness ranging from approximately 7.5–30 nm, and naked mole rat loop formed the longest rod-shaped filaments, approximately 15 nm thick (Figure 8). Variation at even a single site within the β2α2 loop was able to substantially influence overall fibril morphology, as evidenced by fibrils harboring a single-residue change from the bank vole sequence: N170S (Figure 8).

## Discussion

Prion diseases are linked to the conversion of natively folded PrP^C^ into disease-associated PrP^Sc^. Growing evidence suggests the end-point fibril structure of PrP^Sc^ can differ across mammalian species and strains, despite the high similarity of mammalian PrP^C^ structures. The rigid loop hypothesis (Gossert et al., 2005) adds to the collective understanding of the conversion path from PrP^C^ to PrP^Sc^, where sequence differences give rise to subtle, structural changes in PrP^C^ that influence its susceptibility to misfolding and structural conversion. Here, we study the structural features of endpoint structures yielded by mammalian prion segments representing variants of the β2α2 loop of mammalian PrP. These features give rise two types of zipper packing in amyloid structures of the β2α2 loop – clasped and interdigitated – and find that each structure fares differently across chemical environments. Their stabilities vary in the face of high pH or denaturants, while the morphologies of fibrils formed by bank vole PrP^94–178^ appeared altered by sequence variation in its β2α2 loop.

**FIGURE 9:**
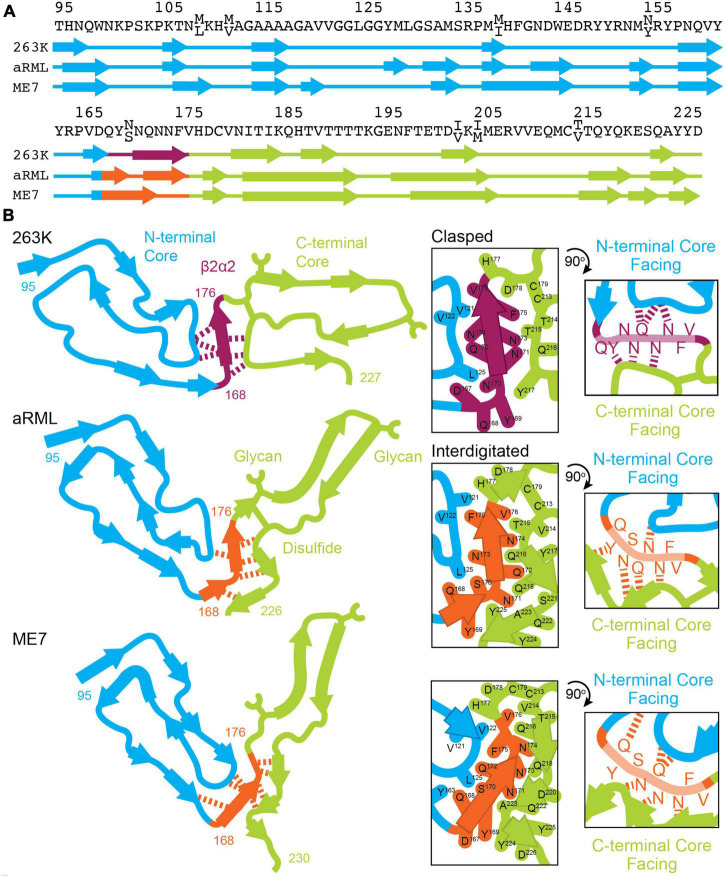
Conformation of β2α2 loop segment in PrP^Sc^ fibril structures. **(A)** Sequence of residues associated with 263K, aRML, and ME7 prion fibrils with numbering according to hamster PrP. **(B)** Schematics of the recently reported structures of PrP^Sc^ determined by single particle cryo electron microscopy (cryoEM) (Hoyt et al., 2021; Kraus et al., 2021; Manka et al., 2021, 2022). Residues of interest are numbered, and hydrogen bonds between the β2α2 loop and N- and C-terminal cores are shown in as dashed lines.

Our collection of amyloid structures formed by β2α2 loop prion segments illustrates two types of interconvertible close packings. The polymorphs we observe appear to be influenced by the pattern of hydrogen bonding adopted by residues in β2α2 loop segments. In clasped zippers, hydrogen bonds are satisfied by residues in the same strand as well as those above and below along a sheet. The interface where these sheets meet is more permissive of varying sequences in a mating strand. In contrast, the interdigitated zipper configuration contains polar residues from one strand that point outward but fit tightly between residues on a mated strand. These residues create an interface more suited to specific residue sizes and biochemical properties on a mating strand, and thus may be more selective. While the differences in local packing we observe for these segments are not predictive of PrP^Sc^ structure, they present atomic resolution views of interconvertible motifs that might stabilize prion fibrils (Figure 9).

As clasped hydrogen bond networks can be rearranged to form interdigitated networks and vice versa, local environment is expected to deeply influence the specific packing observed in PrP^Sc^. Evidence to support this theory is exemplified by structures of mouse aRML and hamster 263K PrP^Sc^, which have interdigitated and clasped structures, respectively, but whose β2α2 loop segment crystal structures are both clasped. This is no surprise, as structures of short amyloid peptides are known to differ from the conformations adopted by the same sequences in the context of full-length fibrils including those of PrP (Wan et al., 2015). In those cases, structures of short segments offer clues to the kind of packing that might be observed in mammalian PrP^Sc^ structures, but may not necessarily define the local or overall fold of a fibril, as the chemical environment differs (Figure 9).

The types of short-range interactions we observe in crystal structures of β2α2 loop peptides are accommodated by some PrP^Sc^ structures, but do not account for all possible modes of amyloid packing accessible by this segment. In some cases, multiple amyloid conformations are accessible to the β2α2 loop, even in the absence of sequence variation. The observation that residue 174 influences the preferred packing of β2α2 loop segments and their corresponding networks of hydrogen bonds is in line with previous reports dictating its importance to prion conversion, alongside residue 170 (Sigurdson et al., 2010). In the 263K and aRML prion structures, the sidechain of N174 forms a hydrogen bond with the backbone of the N-terminal or C-terminal core, respectively. While substitution of S or T for N174 would alter this interaction, the influence of these variations on the overarching fibril fold remains to be seen. The presence of E versus Q at residue 168, S versus N at residue 170, and Q versus E at residue 172 had limited influence on hydrogen bonding networks in amyloid formed by β2α2 peptides alone. Other substitutions including S170N, Y169G, and Y169F also failed to impact packing. Their influence on transmission and protease-resistance could not be explained by our structures of isolated segments, but recent structures of PrP^Sc^ fibrils (Hoyt et al., 2021; Kraus et al., 2021; Manka et al., 2021) indicate that these residues may influence these features through their heterotypic interactions with either N-terminal or C-terminal segments (Figure 9).

As might be anticipated, the weakest aggregates harbored a negatively charged residue at position 168, which prevents the formation of aggregates or readily dissolves existing aggregates at a pH well above the pKa of glutamate (∼4.5). This pH sensitivity is exhibited by structures of the human β2α2 loop segment; the effect cannot be mitigated by introduction of an asparagine at position 170 in place of serine. The native human β2α2 loop segment as well as Y169F and S170N variants paired with E168 were all more sensitive to denaturation by urea and guanidine than structures containing a glutamine at residue 168, except for rabbit β2α2 loop aggregates. It is worth noting that the rabbit β2α2 loop peptide crystallized as a class 1 (face-to-face) (Sawaya et al., 2007) steric zipper with two unique interfaces between mated sheets, whereas all other peptides investigated in stability experiments crystallized as class 2 (face-to-back) steric zippers with a single unique interface between mated sheets. This raises an important structural context not provided by these peptide crystal structures, that of heterotypic interactions. These interactions are the norm in full-length amyloid fibrils and undoubtedly impose strong constraints on the local configurations of residues adopted by various segments, including the β2α2 loop.

## Conclusion

With various atomic structures of β2α2 loop segments of mammalian PrP, we interrogate the impact of sequence variation in this critical region of the protein on amyloid packing and stability. Sequence variation introduced into the β2α2 loop of a bank vole PrP background to match the sequences of other mammalian prions yielded readily distinguishable polymorphs that could be classified based on sequence. Our own observations of differences in PrP fibril morphology induced by sequence variants in the β2α2 loop demonstrate the important influence of the loop on fibril structure. In recently published structures of PrP^Sc^ fibrils, this segment represents a bridge linking two halves of each fibril layer. As such, it represents a critical joint in the overall fold of some PrP^Sc^ fibrils.

## Materials and methods

### Crystallization

All peptides were synthesized and purified by HPLC to 98% purity by Genscript. All peptides were dissolved in water at the concentration listed in Table 1 with the exception of naked mole rat and rabbit/pig/guinea pig 168-176, which were dissolved in 1% and 4% DMSO, respectively. Peptides were then either added directly to 96-well hanging-drop broad screen crystallization trays or filtered using a 0.22 micron filter according to Table 1. Some conditions produced crystals that could be readily identified by light microscopy while others produced aggregates that could be either crystalline or fibrillar. In the absence of clear crystalline hits, conditions with potential crystals were screened by transmission electron microscopy (TEM). Aggregates with crystalline features were also screened for diffraction quality by TEM. Initial hits containing either crystals or near-crystalline aggregates were then optimized *via* 24 well hanging-drop vapor diffusion experiments until the final crystallization conditions that yielded the largest monomorphic crystals with the best diffraction patterns observed by TEM were obtained (Table 1). It is worth noting that optimizing crystallization conditions for many of these peptides was not trivial. Subtle changes in peptide preparation and growth conditions impacted crystal morphology and led to incremental improvements in diffraction quality. For some peptides, many rounds of optimization were needed to reach the final condition used for structure determination.

### Expression and purification BvPrP^94–178^ with β2α2 loop substitutions

A gene encoding bank vole PrP residues 94-178, with M109 genotype was purchased from IDT. Four additional constructs, which were identical in sequence except for β2α2 loop substitutions to match elk, naked mole rat, human, and mouse β2α2 loops, were also purchased. Each construct was cloned into a pET24a + derivative vector lacking purification or solubility tags using Gibson assembly cloning between *Nco*I and *Xho*I restriction sites. The sequence of each construct was verified by DNA sequencing (Genewiz). Each construct was transformed into BL21 GOLD (DE3) cells and grown to an optical density at 600nm (OD_600_) of 0.6–0.8 at 37°C while shaking at 180-230rpm. Overexpression was induced by addition of IPTG to 1mM and cells were allowed to continue shaking under the same condition for 4–6 h before being harvested via centrifugation at 8,000g for 10 min. Cell pellets were stored at –80°C until subsequent purification steps were performed.

Purification was carried out as previously described (Wan et al., 2015; Glynn et al., 2020) and reiterated here. Cell pellets were resuspended in 25 mM Tris-HCl pH 8 and 5 mM EDTA (Buffer A) with HALT protease-inhibitor cocktail (Sigma Aldrich). Cells were then lysed using an EmulsiFlex-C3 High Pressure Homogenizer (Avestin) and pelleted via centrifugation at 30,000g for 1 h (rBvPrP^94–178^ with bank vole, elk, naked mole rat, and human loops) or at 10,000g for 40 min (rBvPrP^94–178^ with mouse/cow loop) at 4°C. The supernatant was removed before the pellet was resuspended again in Buffer A and centrifuged under the same conditions to remove any remaining soluble material. This pellet was either immediately processed or stored at –80°C until purification continued the next day. The pellet was next solubilized in freshly made 8 M Guanidine-HCl, 25 mM Tris-HCl pH 8 and 100 mM DTT (Buffer B) before centrifugation for 20 min at 20,000g (rBvPrP^94–178^ with bank vole, elk, and naked mole rat loops) or 35,000g (rBvPrP^94–178^ with human and mouse) based on how readily soluble and insoluble material separated. The supernatant containing solubilized inclusion bodies was filtered using a 0.45 micron filter. 2 ml per injection of filtered sample was injected into an NGC chromatography system (Biorad) and flowed over an Enrich SEC 650 10 × 300 column (Biorad) equilibrated with freshly made 6 M Guanidine-HCl, 12.5 mM Tris-HCl pH 8, 5 mM DTT and 1 mM EDTA (Buffer C).

All proteins eluted partially in the void volume, indicative of multimers or incomplete solubilization, but a majority eluted as a monomer. Monomeric prion protein-containing fractions were pooled and buffer exchanged into 8M urea using a Duoflow chromatography system (Biorad) and a HiTrap Desalting column (GE Healthcare) either the same day or the next day following flash freezing and storage at –80°C. Protein containing fractions were then pooled and concentrated to 3.2–8 mg/ml before being flash frozen using liquid nitrogen and stored at –80°C until use.

### Fibrillization of rBvPrP^94–178^ with β2α2 loop substitutions

In order to control for differences in fibril morphology induced by differences in growth conditions, all proteins were fibrillized under the same conditions. All fibrils were formed at 1 mg/ml in 1M urea, 200 mM NaCl, and 50 mM NaCitrate pH 4 *via* acoustic resonance mixing at 38Hz for a few days.

### Transmission electron microscopy

Crystalline samples were prepared for Transmission electron microscopy (TEM) screening for crystal and diffraction quality as previously described (Gallagher-Jones et al., 2018). In brief, 2–3 μL drops from 24 well crystallization screens were either pipetted and applied directly to a 300 mesh formvar carbon (F/C) grid (Ted Pella) and allowed to incubate for approximately 2 min before excess liquid was wicked away with filter paper. For drops where crystals stuck to the cover slip or aggregates were difficult to pipet, an additional 2–3 μL of well solution was added to the drop to aid in sample removal before application to a grid. Grids were screened on a Thermo Fisher Tecnai 12 microscope for crystal presence and quality before being screened for dry diffraction quality on a Thermo Fisher Tecnai F20. Samples with the highest quality diffraction were used for microED data collection. Fibrillar samples were prepared similarly and as previously described (Glynn et al., 2020).

### Microcrystal electron diffraction sample preparation

Each peptide crystal solution was pipetted to aid in creating a monodisperse solution for grid application. All holey carbon grids (Quantifoil R 2/4, 1/4, 2/1, or 2/2, 200 or 300 mesh Copper, Electron Microscopy Sciences) were glow discharged using a PELCO easiGlow and plunge frozen into liquid ethane using an FEI Vitrobot Mark IV set to 0% humidity after sample application. 1.5μL (EYNNQNNFV, QYSNQNSFV), 1.8 μL (QYSNQNNFV, EFSNQNNFV, QFNNQNNFV), 2μL (YSNQNNF, EYSNQNNFV, QYNNENNFV, QYNNQNSFV), or 2.5 μL (QYSNQNSFV) was applied to the carbon side only (QYSNQNNFV, EYNNQNNFV, EFSNQNNFV, QFNNQNNFV, QYSNQNNFV) or both sides (YSNQNNF, EYSNQNNFV, QYSNQNSFV, QYNNENNFV, QYNNQNSFV) of the grid. A blot force of 22 was used for all samples except EYNNQNNFV, YSNQNNF and EYSNQNNFV, which used a blot force of 20 (EYNNQNNFV) or 6–10 (YSNQNNF and EYSNQNNFV). Blot times of 15–20 (YSNQNNF, EYSNQNNFV), 18.5 (QYSNQNNFV, EFSNQNNFV, QFNNQNNFV), 20 (EYNNQNNFV), 22 (QYNNENNFV), 24 (QYSNQNSFV), 25 (QYNNQNSFV), or 26 (QYSNQNSFV) were used as diffraction often depended on the state of grid hydration.

### Microcrystal electron diffraction data collection

For the peptides YSNQNNF, EYSNQNNFV, QYSNQNNFV, EYNNQNNFV, EFSNQNNFV, QFNNQNNFV, QYSNQNSFV, QYNNENNFV and GSNQNNF, diffraction patterns were collected under cryogenic conditions using an FEI Tecnai F20 transmission electron microscope operating at 200keV equipped with a bottom mount TemCam-F416 CMOS camera (TVIPS). Diffraction patterns were collected at a detector distance of 730mm or 520mm (QYNNENNFV and GSNQNNF only) with 2 s, 3 s (GSNQNNF only), or 5 s (QYSNQNSFV and GSNQNNF only) exposures while continuously rotating at 0.3 degrees per second, 0.25 degrees per second (QYSNQNSFV and QYNNENNFV only), or 0.2 degrees per second (GSNQNNF only). For QYNNQNSFV, diffraction patterns were also collected under cryogenic conditions, but using a Thermo Fisher Talos Arctica operating at 200keV equipped with a Thermo Fisher CetaD CMOS detector. Diffraction patterns were collected at a detector distance of 750mm with 3 s exposures while the stage was continuously rotated at 0.3 degrees per second.

### Microcrystal electron diffraction data processing

Diffraction images for all peptides were indexed and integrated in XDS (Kabsch, 2010) with the best datasets contributing to the final solutions merged in XSCALE (Kabsch, 2010). All datasets were of sufficient quality at high-resolution to yield unambiguous direct methods solutions and ranged in resolution from 0.85 to 1.05Å. Atomic models were built into Coulomb potential maps in Coot (Emsley et al., 2010) and refined in either PHENIX (Adams et al., 2010) (EYSNQNNFV, QYSNQNNFV, EYNNQNNFV, QYSNQNSFV, QYNNQNSFV), REFMAC (Murshudov et al., 2011) (EFSNQNNFV, QYNNENNFV, QFNNQNNFV), or a combination of PHENIX, REFMAC, and Buster (Blanc et al., 2004) (GSNQNNF, YSNQNNF). Refinement statistics for all structures are listed in Table 2.

**TABLE 2:** Data collection and refinement statistics.

	EYSNQNNFV (7RVC)	QYSNQNNFV (7RVD)	QYNNQNSFV (7RVI)	QYSNQNSFV (7RVG)	EYNNQNNFV (7RVE)	EFSNQNNFV (7RVL)	QFNNQNNFV (7RVF)	QYNNENNFV (7RVH)	GSNQNNF (7RVK)	YSNQNNF (7RVJ)
**Data collection**										
No. Crystals Merged	6	8	5	4	3	8	9	6	7	7
Space group	P1	P1	C2	P2_1_	P1	P1	P1	P1	P1	P1
Cell dimensions										
*a*, *b*, *c* (Å)	10.02 4.89 31.33	4.87 10.17 31.29	62.76 4.85 21.52	23.57 4.86 27.70	4.93 10.14 31.56	4.90 10.38 30.26	4.88 10.56 29.98	4.87 10.06 30.66	14.14 4.87 18.15	24.39 4.95 20.86
α, β, γ (°)	90.99 91.43 102.18	94.65 90.73 101.15	90.00 109.14 90.00	90.00 111.21 90.00	94.13 90.59 102.74	90.91 90.82 102.25	93.89 92.38 103.29	94.85 90.26 99.99	93.21 91.02 101.83	86.52 77.16 85.68
Resolution (Å)^a^	10.44 – 1.00 (1.03 – 1.00)	10.39 – 1.00 (1.03 – 1.00)	10.76 – 1.05 (1.08 – 1.05)	7.18 – 1.00 (1.03 – 1.00)	9.86 – 0.85 (0.87 – 0.85)	10.14 – 1.00 (1.00 – 1.03)	10.24 – 1.00 (1.03 – 1.00)	7.98 – 0.90 (0.93 – 0.90)	13.84 – 1.00 (1.03 – 1.00)	11.87 – 1.00 (1.03 – 1.00)
*R*_merge_,	0.187 (0.432)	0.167 (0.514)	0.287 (0.589)	0.204 (0.698)	0.185 (0.505)	0.178 (0.363)	0.229 (0.636)	0.197 (0.475)	0.238 (0.430)	0.283 (0.478)
*I/*σ(*I*)	5.56 (2.11)	3.99 (1.33)	5.00 (2.47)	5.72 (2.45)	3.81 (1.22)	5.99 (3.66)	5.33 (2.28)	5.21 (2.56)	5.05 (2.25)	4.49 (1.72)
*CC*_1/2_	0.988 (0.840)	0.988 (0.742)	0.981 (0.931)	0.994 (0.699)	0.985 (0.617)	0.985 (0.940)	0.980 (0.711)	0.988 (0.783)	0.980 (0.862)	0.957 (0.336)
Completeness (%)	97.49 (77.00)	93.74 (94.70)	80.00 (83.50)	74.90 (71.70)	89.80 (43.20)	87.10 (69.70)	94.80 (54.90)	81.00 (82.30)	80.30 (64.70)	85.80 (56.5)
Redundancy	5.2 (4.1)	3.0 (2.4)	8.4 (8.6)	7.4 (6.6)	3.5 (2.4)	5.4 (5.0)	6.2 (5.2)	5.4 (4.9)	6.2 (4.3)	5.5 (2.3)
**Refinement**										
Resolution (Å)	1.00 (1.26 – 1.00)	1.00 (1.26 – 1.00)	1.05 (1.32 – 1.05)	1.00 (1.26 – 1.00)	0.85 (0.97 – 0.85)	1.00 (1.26 – 1.00)	1.00 (1.03 – 1.00)	0.90 (0.92 – 0.90)	1.00 (1.12 – 1.00)	1.00 (1.12 – 1.00)
No. reflections	3031 (167)	2977 (215)	2612 (187)	2669 (181)	4694 (161)	2737 (163)	2938 (123)		2076 (123)	4309 (204)
*R*_work_	0.163 (0.174)	0.201 (0.199)	0.227 (0.212)	0.218 (0.259)	0.218 (0.274)	0.192 (0.196)	0.221 (0.336)	0.222 (0.302)	0.202 (0.208)	0.226 (0.222)
*R*_free_	0.168 (0.188)	0.250 (0.257)	0.265 (0.264)	0.220 (0.277)	0.266 (0.331)	0.211 (0.213)	0.251 (0.252)	0.235 (0.254)	0.238 (0.222)	0.233 (0.236)
No. atoms										
Protein	144	147	79	144	148	143	148	148	97	224
Ligand/ion (specify/describe)	0	0	6 (NaCacodylate)	0	0	0	0	0	8 (ZnAcetate)	0
Water	1	0	3	4	2	1	2	2	6	8
*B* factors										
Protein	9.09	8.49	3.33	5.41	23.60	7.95	6.25	4.48	4.95	3.57
Ligand/ion		–	9.47	–	–	–	–	–	8.71	–
Water	20.04	–	9.01	11.46	22.27	24.20	21.24	12.64	14.52	4.40
R.m.s. deviations										
Bond lengths (Å)	0.017	0.008	0.011	0.011	0.013	0.010	0.008	0.009	0.010	0.010
Bond angles (°)	1.607	0.920	1.086	0.844	1.106	1.019	1.662	1.306	0.940	0.910

^a^Values in parentheses are for highest-resolution shell.

### Fibril diffraction of mammalian β2α2 loop peptide crystals or fibrils

Crystalline (QYNNQNNFV, QYSNQNNFV, EYSNQNNFV, QYSNQNSFV, QYNNQNSFV, QFNNQNNFV, EFSNQNNFV) or fibrillar (QYNNQNTFV, RYSNQNNFV, QYSSQNNFV) aggregates were centrifuged at low speed, had growth buffer removed via pipetting, and subjected to addition of a smaller volume of water repeatedly in order to remove excess salts that would compromise fibril diffraction patterns. The concentrated aggregate solution was then applied between two blunted capillaries and allowed to dry. Additional aggregate solution was applied repeatedly until a sufficiently sized aggregate bundle was achieved. All samples with the exception of QYSNQNSFV were allowed to dry before being diffracted with the cryo-stream pointed away from the sample using a 5 min exposure to a FRE + rotating anode generator with VARIMAX HR confocal optics producing Cu K-α radiation (Rigaku, Tokyo, Japan). Diffraction patterns were collected using a RIGAKU R-AXIS HTC imaging plate detector at a distance of 156 mm from the x-ray source. For QYSNQNSFV, small sample volumes prevented complete removal of all excess salts. To minimize salt rings in diffraction patterns, QYSNQNSFV aggregates were diffracted while wet with 50% glycerol applied to the aggregate immediately before diffraction with the cryo-stream directed at the sample.

### Chemical denaturation of mammalian β2α2 loop crystals

Each peptide was allowed to form crystalline aggregates according to the conditions given in Table 1. These solutions were treated similarly to the previously described bank vole β2α2 loop crystals (Gallagher-Jones et al., 2018). Using a high concentration of crystalline material, all crystals were diluted 4x into a final concentration solution of 0.5–4.5M guanidinium-HCl, 0.5–6M urea, 0.75M HCl, MES pH 2.5, NaAc pH 4.5, MES pH 6, Tris-HCl pH 8.5 or 10, MES pH 11.5, NaOH, or water. After mixing with buffers of varying pH, each solution was tested with pH paper to ensure the desired pH had been reached. Since for the rabbit segment, the crystallization buffer reacted with low pH solutions, its crystals were centrifuged at low speed to pellet crystalline material. The supernatant containing the crystallization buffer was removed and replaced with an equal volume of water, which did not cause the crystals to dissolve. Absorbance was used as a proxy for aggregate content, and an absorbance spectra from 250 to 700nm was recorded using a Nanodrop One (Thermo) within a few minutes of mixing crystals with each solution. Each measurement was made in triplicate. A solution consisting of 0.01% w/v latex spheres suspended in water was used as an absorbance control. Absorbance readings for each crystal solution were scaled by setting the untreated crystals to a relative optical density at 600nm of 1, thus readings shown in Figure 6 are absorbance level relative to untreated crystals of the same type.

## Data availability statement

Atomic coordinates and structure factors for all MicroED structures have been deposited in wwPDB under the following accession codes: 7RVC (EYSNQNNFV), 7RVD (QYSNQNNFV), 7RVE (EYNNQNNFV), 7RVF (QFNNQNNFV), 7RVG (QYSNQNSFV), 7RVH (QYNNENNFV), 7RVI (QYNNQNSFV), 7RVJ (YSNQNNF), 7RVK (GSNQNNF), and 7RVL (EFSNQNNFV). Other structures that are described in this work but previously published can be found under PDB accession codes 2OL9 (SNQNNF) (Sawaya et al., 2007), 6AXZ (QYNNQNNFV) (Gallagher-Jones et al., 2018), and 3FVA (NNQNTF) (Wiltzius et al., 2009). Source data for all figures and files is available from the authors upon request.

## Author contributions

JR directed the work. JR, EH, JM, and CG grew, evaluated, and optimized crystals. JR, CG, and EH collected data. JR, CG, EH, MG-J, JM, and CS analyzed the data. CG, CS, and JR wrote the article with input from all authors. All authors contributed to the article and approved the submitted version.
